# Exploring the efficacy of melodic intonation therapy with Broca’s aphasia in Arabic

**DOI:** 10.4102/sajcd.v65i1.567

**Published:** 2018-05-31

**Authors:** Khalid G. Al-Shdifat, Jawdat Sarsak, Fatoon A. Ghareeb

**Affiliations:** 1 Department of Rehabilitation Sciences, Faculty of Applied Medical Sciences, Jordan University of Science and Technology, Jordan; 2 Amman Center for Speech-Language and Swallowing, Amman, Jordan

## Abstract

**Background:**

Even though the efficacy of melodic intonation therapy (MIT) with persons with aphasia (PWA) has been explored in different languages, the efficacy of MIT with Arabic-speaking PWA has never been explored.

**Aims:**

To explore the efficacy of MIT, adapted to Arabic, in promoting the expressive abilities of a 70-year-old Jordanian Arabic-speaking male subject with severe Broca’s aphasia 3 months post-onset.

**Methods:**

An 8-week MIT therapy programme with tapping (1.5 h daily, 6 days a week) was used in a multiple baseline design across two types of trained phrases (i.e. automatic and self-generated phrases). Outcome measures included accuracy of production of trained (at the end of each session) and untrained phrases (at the end of each week). Pre- and post-treatment measures used, were the bilingual aphasia test, the American Speech-Language Hearing Association Functional Assessment of Communicative Skills, the communicative effectiveness index and the American Speech and Hearing Association Quality of Communication Life Scale. Accuracy of production for the trained and untrained phrases was also measured 2 weeks and 4 weeks after the treatment programme was finished.

**Results:**

The patient, (MK), improved his expressive productions post-treatment in automatic and self-generated phrases. Automatic phrases exceeded the established 75% accuracy criterion, whereas the self-generated phrases reached criterion and remained constant at follow-up. Moreover, MK gradually started improving on the generalisation stimuli, once the treatment on the self-generated phrases started and maintained the gains 2 weeks and 4 weeks post-treatment.

**Conclusion:**

MIT appears to be a viable treatment option for Jordanian Arabic-speaking persons with Broca’s aphasia. However, more research is needed with larger groups of Jordanian Arabic-speaking persons with Broca’s aphasia to provide more support to the present findings. Moreover, future studies might focus on the efficacy of MIT with persons with Broca’s aphasia from different Arab countries and from countries where Arabic is part of the multicultural structure like South Africa and other countries on the African continent.

## Introduction

The global burden of aphasia estimates are 25.7 million individuals (Feigin et al., 2015). It is worth noting that between 22.7 and 250 per 100 000 people have a stroke every year, while survival rates are becoming higher, the prevalence rate falls between 508 and 777 per 100 000 people (El-Hajj et al., 2016). This high prevalence rate underscores the importance of research studies in general and intervention studies to try to alleviate the burden placed on stroke survivors and their families. Nonetheless, studies related to aphasia in Arabic have previously looked at the nature of the speech of Arabic-speaking persons with aphasia (PWA), for example, Albustanji, Milman, Fox and Bourgeois (2013) looked at the nature of morphosyntax of Jordanian Arabic-speaking PWA. Adam (2014) compared the noun to verb production abilities in the speech of Palestinian Arabic-speaking PWA. In addition, Freidmann (2006) examined the speech of two Palestinian Arabic speakers and a group of Hebrew-speaking persons with Broca’s aphasia. She compared their accuracy of producing ‘yes/no’ questions to wh-questions in spontaneous speech and in an elicitation task. Hence, it seems that none of the previous studies has examined the effect of any intervention programme on improving the speech and language abilities of Arabic-speaking PWA. Therefore, using an intervention programme like melodic intonation therapy (MIT) with Arabic-speaking PWA might help them regain functional speech and might provide more support to the efficacy of MIT in general. Moreover, most studies that examined the effectiveness of MIT have used it with patients in the chronic phase. Thus, examining the effectiveness of MIT in the subacute phase is of importance (Hurkmans et al., 2012).

For many years, clinicians have reported that Broca’s aphasic subjects are good at singing some words where they are articulated at a slow rate, but they cannot produce them at a normal rate (Brust, 2003; Schlaug, Marchina & Norton, 2008; Schlaug, Norton, Marchina, Zipse, & Wan, 2010). Because the right hemisphere is oriented to music and prosody, this led to the development of MIT to utilise the right hemisphere’s ability for melody and music in the treatment of severely non-fluent aphasic patients to recover speech (Albert, Sparks, & Helm, 1973; Norton, Zipse, Marchina, & Schlaug, 2009; Ozdemir, Norton, & Schlaug, 2006; Racette, Bard, & Peretz, 2006; Schlaug et al., 2008; Sparks, Helm, & Albert, 1974; Sparks & Holland, 1976; Wan, Rüber, Hohmann, & Schlaug, 2010; Wilson, Parsons, & Reutens, 2006). However, recent research suggests different mechanisms that might come to function when using MIT (i.e. brain neuroplasticity in utilising the right hemisphere for language, utilisation of the mirror neuron system to improve motor speech production, shared processing pathways of music and language and the pleasurably engaging way singing can help PWA express themselves) with PWA (Merrett, Peretz, & Wilson, 2014). Although MIT is recommended for subjects with severe non-fluent aphasia to restore their verbal communicative abilities (Ferguson et al., 1994; Norton et al., 2009; Sparks et al., 1974; Sparks & Holland, 1976), it has been used with milder forms of non-fluent aphasia (Hough, 2010; Vines, Norton, & Schlaug, 2011).

MIT mainly focuses on utilising the exaggerated prosody of speech to help non-fluent aphasic subjects improve speech production (Vines et al., 2011). This therapy makes alterations to normal prosody giving higher pitch to stressed syllables on words while keeping the original variation of pitch differences found in the sentence (Sparks & Holland, 1976). Sparks and Holland (1976) were the first to describe MIT which is unlike singing, as it uses a limited range of musical notes by manipulating elements of prosody (i.e. slow tempo, precise rhythm and distinct stress) to help aphasic patients regain speech (Sparks & Holland, 1976). In addition, intensive therapy using MIT is recommended in the treatment of patients with Broca’s aphasia (Ferguson et al., 1994; Schlaug et al., 2008; Wan et al., 2010) which might help patients reach generalisation to untreated stimuli. When non-intensive therapy (e.g. three sessions per week [Hough, 2010]) is used, generalisation to untreated items might not be reached.

When compared to other types of treatment, such as rehearsed and unrehearsed phrase production, combining melody to phrase production in MIT had longer term effects on the production of phrases (Wilson et al., 2006). Moreover, such gains in the production of phrases using MIT were found in other studies (Hough, 2010; Schlaug et al., 2008). Longer term gains are key outcome in the choice of intervention programmes for PWA. The best candidates who might benefit from an MIT programme are Broca’s aphasics who demonstrate better auditory comprehension than verbal abilities, good attention span, motivation and emotional stability (Norton et al., 2009).

The effectiveness of MIT was explored in the treatment of patients with Broca’s aphasia in other languages, such as English (Albert et al., 1973; Hough, 2010; Schlaug et al., 2008; Schlaug et al., 2010; Sparks et al., 1974; Wilson et al., 2006), French (Zumbansen, Peretz, & Hébert, 2014) and Italian (Cortese, Riganello, Arcuri, Pignataro, & Buglione 2015). These studies reported improvements in the speech of their subjects to varying degrees. Even though MIT was explored in these languages, this type of treatment with Arabic Broca’s aphasic patients was never explored. Before discussing an intervention using MIT with Arabic-speaking PWA, it is important to explain the prosody of the Arabic language.

### Intonation

Intonation is usually used for the purposes of manipulating discourse, expressing intents, modifying the sentence grammar and controlling speech register (Bonakdarpour, Eftekharzadeh, & Ashayeri, 2003). Intonation in Arabic is similar to English in using low intonation levels with declaratives in general. However, it seems that Arabic differs from English in its use of low–high intonation contours at the end of interrogative sentences, whereas English uses high-rising intonation contours at the end of questions (de Jong & Zawaydeh, 1999).

#### Stress

Arabic has three basic types of syllables, which are light, heavy and superheavy, and stress usually falls on the heavy or superheavy syllables in Arabic (Watson, 2011). Moreover these three basic syllables have six-syllable structure types. The first syllable is the light syllable and is always made up of a consonant and a short vowel (CV). The second is the heavy syllable and is usually made up of two-syllable structure types, which are a consonant and a long vowel or a diphthong (CVV), or a consonant followed by a short vowel followed by a consonant (CVC). A superheavy syllable is usually made up of three-syllable structure types, namely a consonant followed by a long vowel or a diphthong and a consonant (CVVC), or a consonant followed by a long vowel and two consonants (CVVCC), or a consonant followed by a vowel and two consonants (CVCC) (Halpern, 2009). In Jordanian Arabic, when a word contains a heavy syllable next to the last syllable in a word, stress usually falls on the heavy syllable regardless of the number of syllables in the word. If there is no heavy syllable next to the last syllable in the word or the word does not contain a heavy syllable, the stress usually falls on the syllable that comes next to the last syllable in the word. In addition, if the last syllable in a word contains a long vowel or ends with a consonant cluster, then the stress is placed on that syllable in the word (de Jong & Zawaydeh, 1999).

**Rhythm:** Arabic is stress-timed similar to English (Nespor, Shukla, & Mehler, 2011). However, Arabic does not strictly produce syllable stress at the same phases within phrases like English. In addition, the fundamental frequency (F1) for low vowels in stressed syllables in Arabic is between 10 and 40 Hz, compared to 50 and 100 Hz in English (Tajima, Zawaydeh, & Kitabara, 1999).

Based on the aforementioned characteristics of Arabic prosody, MIT was determined to be used with a Jordanian Arabic-speaking patient (i.e. MK) with Broca’s aphasia taking into account differences from English language. Stressed syllables were produced with a higher pitch, keeping the same stress patterns found in normal spoken Arabic. This study followed Hough (2010) procedures with some modifications, such as intensity of therapy, which was increased, and explicitly signalling the patient to wait to promote inner rehearsal. Thus, the purpose of the current study was to investigate the effectiveness of an MIT programme with a Jordanian monolingual Arabic-speaking patient with Broca’s aphasia, which is to our knowledge the first study to examine an effectiveness of a treatment programme for aphasia with an Arabic-speaking patient.

## Method

### Participant

The participant (MK) in this study was a 70-year-old Jordanian male subject who is a monolingual Arabic speaker with an education level of high school. He is married and self-employed. Moreover, MK and his family gave consent for him to participate in the current study, and he signed consent in the presence of his son to participate in the study. MK was right-handed prior to his illness and was enrolled in the MIT programme 3 months post-onset where he had conventional speech therapy (i.e. picture naming), prior to enrolment in the MIT programme. Magnetic resonance imaging (MRI) and magnetic resonance angiography reports confirmed occlusion of the left internal carotid artery, which affected the middle communicating and the anterior cerebral arteries, resulting in severe Broca’s aphasia. He could only produce few intelligible words, such as yes and no. He also presented with a moderate form of apraxia of speech. Perseveration was also noticed in his attempts to communicate and his speech was markedly laboured. He could follow simple one-step verbal commands and his auditory comprehension was relatively intact as can be seen from the aphasia test results (Table 1). In addition, MK was aware of his communicative problem as he attempted to correct his mis-productions most of the time with repeated trials. He demonstrated many errors when he was asked to write words and phrases.

**TABLE 1:** Pre- and post-treatment scores on the bilingual aphasia test – The Jordanian Arabic Version.

Task	Pre-treatment	Post-treatment	Difference
Spontaneous speech	0	2	2
Comprehension	9	10	1
Pointing (/10)	9	10	1
Simple and semi-complex commands (/10)	7	8	1
Complex commands (/5)	2	2	0
Total (/25)	18	20	2
Verbal auditory discrimination (/18)	14	16	2
Syntactic comprehension (/87)	37	54	17
Semantic categories (/5)	5	5	0
Synonyms (/5)	1	3	2
Antonyms (/5)	8	9	1
Grammatical judgement (/10)	8	9	1
Semantic acceptability (/10)	10	10	0
Repetition of words and nonsense words, and lexical decision (/60)	32	46	14
Sentence repetition (/7)	0	3	3
Series (/3)	0	1	1
Verbal fluency per minute	3	10	7
Naming (/20)	1	6	5
Sentence construction (/5)	0	1	1
Semantic opposites (/10)	0	0	0
Derivational morphology (/20)	0	0	0
Description (/3)	0	0	0
Mental arithmetic (/15)	14	14	0
Listening comprehension (/5)	0	1	1
Reading words and sentences (/20)	0	0	0
Reading paragraph	0	0	0
Copying (/5)	4	4	0
Dictation for words and sentences (/10)	3	6	3
Reading comprehension of words and sentences (/20)	5	12	7
**Total difference**	**-**	**-**	**+67**

*Source*: Paradis, M., & Libben, G. (1987). *The assessment of bilingual aphasia*. Hillsdale, NJ: Lawrence Erlbaum Associates

Because MK was highly motivated for therapy, emotionally stable and had relatively good auditory comprehension, these factors were indicators that MK was a good candidate for the MIT programme.

#### Materials

The Jordanian Arabic Version of the bilingual aphasia test (BAT) (Paradis & Libben, 1987) was used to measure the linguistic abilities of MK. The BAT consists of three parts: part one is related to bilingualism, part two assesses language skills in Arabic and part three assesses translation skills. Part two of the BAT (the language assessment) is formed of 427 items that allow a thorough assessment of language skills and was used with MK in the present study. In addition, the American Speech-Language Hearing Association Functional Assessment of Communicative Skills (ASHA-FACS) (Frattali, Thompson, Holland, Wohl, & Ferketic, 1995) was used to assess MK’s performance across four domains: (1) social communication; (2) communication of basic needs; (3) reading, writing and number concepts; and (4) daily planning. The ASHA-FACS consists of 43 items that are completed by the clinician after at least three meetings with the patient. It uses a 7-point Likert scale starting with 1 meaning total absence of a skill, even with assistance provided, and 7 meaning total presence of a skill which is performed independently with no prompting to communicate and/or assistance. Moreover, the ASHA-FACS pre-treatment ratings were not made available to the rater in the post-treatment ratings. The Communicative Effectiveness Index (CETI) (Lomas et al., 1989) was used to measure the change in MK’s functional communication ability, as perceived by his close family (i.e. son). MK’s son was asked to complete the CETI in order to get a clear picture of the change in MK’s functional communication ability, based on his son’s observation as his son spent most of the time with him at work and at home. The American Speech and Hearing Association Quality of Communication Life Scale (ASHA-QCL) (Paul et al., 2004) provides information on the effect of aphasia on the affected individual’s social life, self-confidence like confidence in one’s ability to communicate and roles (e.g. family roles). It is a self-report measure and each item consists of a five-point vertical visual analogue scale. MK’s self-rating and the change in scores on the ASHA-QCL pre- and post-treatment scores are recorded in Table 4. To avoid bias, a clinician who was blind to the study was asked to administer the assessments pre- and post-treatment.

**Stimuli:** In all, 55 phrases were selected as treatment stimuli to be used with MK in the present study. The stimuli were divided into 20 automatic phrases, 20 self-generated phrases and 15 (self-generated) generalisation stimuli. To ensure functionality of the phrases selected for therapy, MK’s son and spouse were asked to write down the most important phrases he used at home, work, etc. that were important for his daily communicative needs. The family wrote approximately 100 phrases, which were then classified into automatic or self-generated phrases. The three researchers and a clinician (four raters) completed this process. The phrases that received the same classification by the four raters were used in the intervention programme, whereas the phrases that were in disagreement were not included. A total of 95 phrases received the same classification by three out of four to four out of four raters. Then 70 phrases were included in the study, which had the same rating by the four raters. Of the 70 phrases, 45 phrases were classified as self-generated, while 25 were classified as automatic phrases. After that, we asked MK’s son and wife to select 35 out of the 45 self-generated phrases that they considered most important to MK’s daily communicative needs and to select 20 of the 25 automatic phrases. The researchers used such selection criteria to ensure functionality of the phrases chosen for therapy to help MK make use of family-centred care and to restore daily communication routines.

Examples of stimuli used in the present study:

**Table:** 

Automatic phrases: Good night تصبح على خير Good job يعطيك العافيه No problem بسيطه Forgive me سامحني	Self-generated phrases: Going out for prayer طالع اصلي I want to take a shower داخل اتحمم Leaving to work طالع على الشغل Get my car ready جهز سيارتي

**Procedure:** A multiple baseline design across the two types of phrases (i.e. automatic and self-generated phrases) was used. Because MK had speech and writing difficulties, his son and spouse wrote down the phrases and these were read to MK by one of the researchers for agreement. He agreed with his wife and son on all the phrases. Then, the researchers classified the phrases as automatic or self-generated phrases. Self-generated phrases were defined as phrases used to communicate thought involving semantic processing, while automatic phrases were defined as overlearned forms, involving phonological processing, following definition of propositional (self-generated) and non-propositional (automatic) phrases proposed by Lum and Ellis (1994). Nonetheless, all the phrases used were relevant to the patient’s daily communicative needs, which emphasise the functionality of the phrases (Hough, 2010; Sparks & Holland, 1976).

A condensed eight-step continuum (Rosenbek, 1985) of the four-level model described by Sparks and Holland (1976) was used in the current study. The reason for choosing this eight-step continuum is because we wanted MK to use both types of phrases in his everyday communication. The model described by Sparks and Holland (1976) uses short phrases in the first two levels to familiarise a non-fluent aphasic person to intoned speech. In the first step of the adopted eight-step continuum, the client was required to tap the hummed and then intoned utterance with his left hand while he just listened to the clinician humming and intoning each phrase. The clinician waited 5 s after each time he hummed and intoned the phrase and then repeated the procedure as he signalled the client to wait. This allowed for establishing inner rehearsal (Norton et al., 2009) and to control MK’s verbal perseverations. MK’s perseverations were noticeable and we believed that controlling them would help him focus on the targeted stimuli. Stark (2011) defines perseveration as the inappropriate repetition of a previously produced stimulus. The reason why people with Broca’s aphasia perseverate on verbal production might be that they can notice their errors and thus try to correct them (Stark, 2011). In step two, the client was required to intone the utterance in unison with the clinician while he tapped the rhythm with his left hand. If the client had difficulty tapping along, the clinician tapped the rhythm. In step three, the clinician started the production of the phrase along with the client and then faded out from unison production, keeping the visual cues allowing the client to complete the intoned phrase by himself. If the client could not produce the phrase at a certain step, the phrase was recycled using the previous step. In step four, the clinician modelled each intoned phrase and waited 5 s and at the same time signalled the client to wait; and then he asked the client to imitate the intoned phrase. In step five, the client produced each phrase in unison with the clinician using exaggerated inflection and gradually approximating normal speech prosody. In step six, the clinician kept the visual cues while withdrawing from unison production. In step seven, the clinician produced each phrase using normal prosody and then waited 5 s and then asked the client to repeat the phrase using normal prosody. In step eight, the client was required to produce the phrase in response to the clinician’s question. All of the 20 phrases were targeted during each session at each step of the treatment.

The dependent variable was accuracy of phrase production. Thus, if each word in the phrase was produced accurately and was intelligible, the phrase production was marked accurate. Whether the client produced the phrase with normal prosody or with exaggerated inflection, the phrase was considered accurate. The intervention started with the automatic phrases, deeming these phrases easier for the client to produce. In addition, they were targeted first to examine if he would generalise to the self-generated phrases and to the untreated probes (generalisation stimuli). Baseline data were taken daily for the self-generated phrases as the intervention programme proceeded with the treatment of the automatic phrases. Once the criterion for the automatic phrases was reached, treatment of the self-generated phrases was started and maintenance checking for the automatic phrases was done daily. Probing the generalisation of untreated stimuli was continued at the end of each week. Because our intervention programme was 8 weeks long, we adopted Hough’s (2010) criteria of 75% accuracy so we could move from the automatic to the self-generated phrases within the time frame for the study.

**Analysis:** The data were analysed using the Welch’s two-samples *t-*test to compare the automatic phrase scores to the maintenance and follow-up phrase scores. It was also used to compare the self-generated phrase scores on baseline to the follow-up. This test was used as a result of the skewed nature of the data and because the data were expected to be of unequal variances. In addition, the Durbin–Watson test was utilised to check that the data points had no autocorrelation.

## Results

The results seem to indicate that MK has benefitted from MIT. MK’s post-treatment BAT scores have improved compared to his pre-treatment BAT scores (Table 1). MK’s rating on the ASHA-FACS has improved as well (Table 2); his score prior to intervention was three and rose to six post-intervention. The CETI pre- and post-treatment ratings of MK by his son show that his son believed his father improved post-treatment compared to pre-treatment. The difference between the pre- and post-treatment rating was +47.9 (Table 3).

**TABLE 2:** Pre- and post-treatment ratings on the American Speech-Language Hearing Association Functional Assessment of Communicative Skills.

Dimension	Pre-treatment	Post-treatment
Social communication	3.0	6.0
Communication of basic needs	4.0	6.5
Reading, writing and number concepts	2.0	6.0
Daily planning	3.0	5.5
Overall score	3.0	6.0

*Source*: Frattali, C., Thompson, C., Holland, A., Wohl, C., & Ferketic, M. (1995). *The American Speech-Language-Hearing Association functional assessment of communication skills for adults (ASHA FACS)*. Rockville, MD: ASHA

Note: Ratings are based on 1–7 scale.

**TABLE 3:** Communicative effectiveness index pre-treatment and post-treatment ratings of MK by his son and difference scores.

Statement	Pre-treatment	Post-treatment	Difference
Getting somebody’s attention	3.5	6.0	2.5
Getting involved in group conversations that are about him or her	2.0	5.7	3.7
Giving yes and no answers appropriately	5.0	8.0	3
Communicating his or her emotions	2.3	5.9	3.6
Indicating that he or she understands what is being said to him or her	4.6	9.1	4.5
Having coffee time visits and conversations with friends and neighbours	0.9	3.0	2.1
Having a one-to-one conversation with you	1.0	5.0	4.0
Saying the name of someone whose face is in front of him or her	1.4	3.6	2.2
Communicating physical problems such as aches and pains	1.1	4.7	3.6
Having a spontaneous conversation	0.6	2.4	1.8
Responding to or communicating anything without words	7.0	8.0	1.0
Starting a conversation with people who are not close family	3.0	6.0	3.0
Understanding writing	2.9	5.6	2.7
Being part of a conversation when it is fast with a number of people involved	1.0	3.0	2.0
Participating in a conversation with strangers	0.7	2.6	1.9
Describing or discussing something in depth	0.5	1.5	1.0
Overall difference in score	-	-	47.9†

*Source*: Lomas, J., Pickard, L., Bester, S., Elbard, H., Finlayson, A., & Zoghaib, C. (1989). The communicative effectiveness index: Development and psychometric evaluation of a functional communication measure for adult aphasia. *Journal of Speech and Hearing Disorders, 54*, 113–124. https://doi.org/10.1044/jshd.5401.113

† , Scores are based on a 10-cm visual analogue scale used in the communicative effectiveness index; scale is 1–10 converted to a score of 10.

**FIGURE 1:**
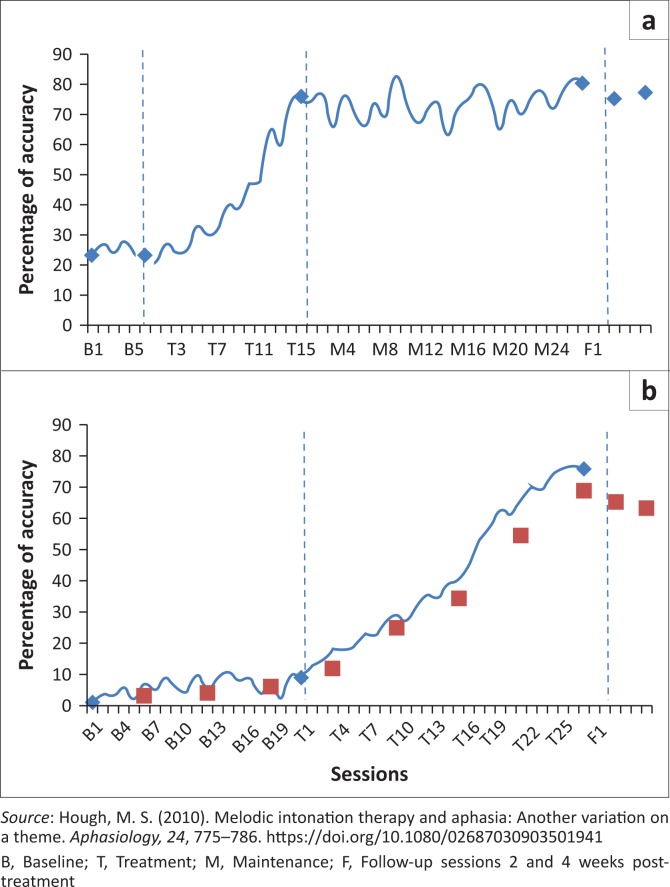
Percentages of accuracy for the automatic, self-generated and generalisation phrases used in the current study: (a) presents treatment of the automatic phrases; (b) presents treatment of the self-generated phrases measured daily and the generalisation stimuli measured at the end of every week.

MK’s quality of life score measured using the ASHA-QCL scale reveals an increase post-treatment of +25.9 compared to his score pre-treatment (Table 4). There was a gradual improvement of MK’s verbal productions as treatment progressed (Figure 1). He exceeded the criterion (i.e. 75%) for the automatic phrases and kept the gains even during the maintenance and follow-up phases. During training on the automatic phrases and even when the baseline extended for more than 2 weeks for the self-generated phrases, there was no noticeable improvement on the production of this type of phrases (Figure 1). However, MK reached criterion, that is, 75% accuracy for the self-generated phrases after treatment was initiated for those phrases.

**TABLE 4:** MK’s scores pre- and post-treatment scores† on the American Speech-Language Hearing Association Quality of Communication Life scale.

Statement	Pre-treatment	Post-treatment	Difference
‘I like to talk to people.’	2.0	4.0	2.0
‘It’s easy for me to communicate.’	1.0	3.0	2.0
‘My role in the family is the same.’	2.0	4.0	2.0
‘I like myself.’	3.0	4.0	1.0
‘I meet the communication needs of my job or school.’	1.0	3.0	2.0
‘I stay in touch with family and friends.’	2.0	4.0	2.0
‘People include me in conversations.’	3.0	4.3	1.3
‘I follow news, sports and stories on TV or movies.’	1.0	2.0	1.0
‘I use the telephone.’	1.0	2.5	1.5
‘I see the funny things in life.’	1.0	3.0	2.0
‘People understand me when I talk.’	1.0	3.0	2.0
‘I keep trying when people don’t understand me.’	4.0	4.5	0.5
‘I make my own decisions.’	4.0	4.3	0.3
‘I am confident that I can communicate.’	3.0	4.6	1.6
‘I get out of the house and do things.’	2.0	3.7	1.7
‘I have household responsibilities.’	2.0	3.1	1.1
‘I speak for myself.’	2.0	4.0	2.0
‘In general, my quality of life is good.’	2.0	4.0	2.0
**Total difference score**	-	-	25.9†

*Source*: Paul, D., Frattali, C., Holland, A., Thompson, C., Caperton, C., & Slater, S. (2004). *ASHA Quality of Communication Life Scale (QCL)*. Rockville, MD: American Speech-Language-Hearing Association

† , Scores are based on a 5-point scale (1–5).

Welch’s *t*-test was used to check if the differences between baselines and post-treatments were significant for the two types of phrases targeted in therapy. The Welch’s *t*-test for the automatic phrases revealed a significant difference comparing MK’s scores in the baseline to the maintenance and post-treatment scores (*t* = 38.118; *df* = 18.825; *p* = 0.000). In addition, there was a significant difference for the self-generated phrases when the baseline performance was compared to post-treatment (*t* = 83.329; *df* = 6.324; *p* = 0.000). The data were also examined for autocorrelation using the Durbin–Watson test; the Durbin–Watson test showed that there was no autocorrelation between data points for the automatic and self-generated phrases. The Durbin–Watson score for the automatic phrases was 1.95 and 1.72 for the self-generated phrases.

## Discussion

The purpose of the current investigation was to examine the efficacy of MIT with a Jordanian Arabic-speaking PWA. The results of the study show that MIT seems a viable treatment option for Jordanian Arabic aphasic patients. Indeed, the benefit of rhythm and pitch elicited the strongest generalisation effect, both to non-trained stimuli and connected speech, compared to normally spoken and to rhythmic therapy alone (Zumbansen et al., 2014). MK’s verbal ability seems to have improved when treatment using MIT was initiated. This improvement was evident from the score change that can be seen on the BAT. Nonetheless, one administration of the BAT would not allow for a solid conclusion, so future studies might consider multiple administrations of the instrument prior to and during therapy. MK’s production accuracy improved on the automatic and self-generated phrases; the improvement was gradual. MK’s accuracy producing the self-generated phrases did not start to improve until they were targeted in therapy. Moreover, his accuracy on the generalisation stimuli started to improve gradually when therapy started targeting the self-generated phrases but not when the automatic phrases were targeted in therapy. This might be as a result of the nature of automatic phrases, as they are considered overlearned forms. This is consistent with Stahl, Henseler, Turner, Geyer and Kotz (2013) where they found that their subjects did not generalise beyond formulaic phrases when singing and rhythmic therapy were used. Because MIT utilises rhythm and pitch, improvement on phrase production was observed in the current study.

There are some reasons that might explain why MK improved in therapy using MIT in Arabic. Firstly, his improvement might be because he had better auditory comprehension than verbal abilities; he had good attention span; he was motivated to be enrolled in therapy; and he was emotionally stable (Helm, Nicholas, & Morgan, 1989). Secondly, the intensity of therapy might have resulted in MK’s improvement and his reaching the set accuracy level for both types of phrases. He received six sessions per week and each session was 1.5 h long. Such intensive therapy using MIT is recommended for PWA (Schlaug et al., 2008; Wan et al., 2010). Hough (2010) reported that while her aphasic patient reached criterion level for the automatic phrases, he did not reach criterion for the self-generated phrases. Her patient might have reached criterion for the automatic phrases because these types of phrases are overlearned forms and do not need much effort. On the other hand, her patient did not reach criterion for the self-generated phrases because these phrases carry thought, and unlike automatic phrases, they are not overlearned (Lum & Ellis, 1994). Reaching criteria for the two types of phrases might also be attributed to the functionality of communication of the two types of phrases (Hough, 2010; Sparks & Holland, 1976) by asking MK’s son and spouse to write the most important phrases for the patient in his daily interactions to ensure functionality of the targeted phrases in therapy.

Another reason that might account for MK’s improvement and his attainment of accuracy criteria for the two types of phrases is the nature of Arabic language. Arabic language speakers might employ intonation in some aspects of their speech more than English language speakers. Intonation can vary meanings of the same utterance (O’Connor, 1980; Halliday, 1970). Speakers of colloquial Arabic or everyday Arabic tend to employ intonation in their speech most of the time to convey different communicative purposes. For instance, speakers of colloquial Arabic do not use a question word to form ‘yes/no’ questions; they use intonation instead. This might explain why MK reached criterion and maintained the gain at follow-up. However, this is speculative because there are no functional MRI studies on Arabic speakers to corroborate this assumption. Support for this notion might come from Friedman’s (2006) finding in that ‘yes/no’ questions were highly accurate in spontaneous speech and in an elicitation task, compared to wh-questions in two Palestinian Arabic-speaking aphasic patients. The homologous areas of speech-motor and language in the right part of the brain might be equipped with the ability to take over the speech and language tasks of the damaged areas in the left hemisphere (Rosen et al., 2006; Schlaug et al., 2008).

The design of the MIT programme may have promoted improvement in MK’s speech; our study targeted automatic phrases as MK did not show improvement on the self-generated phrases until MIT targeted those phrases.

### Limitations and future directions

The current study was limited to only one Jordanian Arabic speaker with aphasia in one Arabic-speaking country, which is Jordan. The study was limited to the use of one variation of MIT, which limited the scope of comparing the efficacy of other variations of MIT (e.g. use of MIT with transcranial direct current stimulation). Future intervention studies using MIT with Arabic PWA might consider enrolling larger numbers of patients. They might also consider implementing intervention studies in different Arabic-speaking countries to check the efficacy of these interventions with speakers of different Arabic dialects. Furthermore, future studies with Arabic-speaking PWA might want to consider using different variations of MIT programmes to check their efficacy in Arabic. They might also want to consider the variation of changing treatment stimuli to ‘yes/no’ interrogative questions and gradually changing them to declarative statements as accuracy of their production increases.

## Summary and conclusion

This is the first intervention study to test the efficacy of a treatment programme for Arabic-speaking PWA. An MIT programme was used with a Jordanian Arabic-speaking patient with severe Broca’s aphasia. The subject in the current study benefitted from MIT and his accuracy improved on three types of phrases, namely automatic phrases, self-generated phrases and generalisation phrases (untreated phrases). Because no research has explored the effectiveness of aphasia treatments with Arabic PWA, the results of the current study using MIT might form a stepping stone in promoting more research on the efficacy of MIT treatments with Arabic-speaking PWA.
